# Nontypeable *Haemophilus influenzae* released from biofilm residence by monoclonal antibody directed against a biofilm matrix component display a vulnerable phenotype

**DOI:** 10.1038/s41598-023-40284-5

**Published:** 2023-08-10

**Authors:** Kathryn Q. Wilbanks, Elaine M. Mokrzan, Theresa M. Kesler, Nikola Kurbatfinski, Steven D. Goodman, Lauren O. Bakaletz

**Affiliations:** 1 Center for Microbial Pathogenesis, Abigail Wexner Research Institute at Nationwide Children’s Hospital, Columbus, OH 43205 USA; 2 Department of Pediatrics, The Ohio State University College of Medicine, Columbus, OH 43205 USA

**Keywords:** Microbiology, Biofilms

## Abstract

Bacterial biofilms contribute significantly to pathogenesis, recurrence and/or chronicity of the majority of bacterial diseases due to their notable recalcitrance to clearance. Herein, we examined kinetics of the enhanced sensitivity of nontypeable *Haemophilus influenzae* (NTHI) newly released (NRel) from biofilm residence by a monoclonal antibody against a bacterial DNABII protein (α-DNABII) to preferential killing by a β-lactam antibiotic. This phenotype was detected within 5 min and lasted for ~ 6 h. Relative expression of genes selected due to their known involvement in sensitivity to a β-lactam showed transient up-regulated expression of penicillin binding proteins by α-DNABII NTHI NRel, whereas there was limited expression of the β-lactamase precursor. Transient down-regulated expression of mediators of oxidative stress supported similarly timed vulnerability to NADPH-oxidase sensitive intracellular killing by activated human PMNs. Further, transient up-regulated expression of the major NTHI porin aligned well with observed increased membrane permeability of α-DNABII NTHI NRel, a characteristic also shown by NRel of three additional pathogens. These data provide mechanistic insights as to the transient, yet highly vulnerable, α-DNABII NRel phenotype. This heightened understanding supports continued validation of this novel therapeutic approach designed to leverage knowledge of the α-DNABII NRel phenotype for more effective eradication of recalcitrant biofilm-related diseases.

## Introduction

Bacterial biofilms contribute significantly to the pathogenesis of acute and chronic infections^1^, as well as to the recurrence and recalcitrance of many diseases to treatment^2^. Common diseases in which biofilms play a key role include otitis media, chronic obstructive pulmonary disease, periodontitis, and cystic fibrosis, among many others^3–5^. Canonical biofilm tolerance is attributable to multiple factors, but importantly, and from the standpoint of disease resolution, bacteria within a biofilm are highly resistant to both antibiotics and host immune effectors^6–8^. Indeed, biofilm-resident bacteria are up to 1000 times more resistant to conventional antibiotics relative to their planktonically grown counterparts^9–11^. Further, infections that involve biofilms are costly; it is estimated that the global economic burden of biofilms related to medical and human healthcare costs was approximately $387 billion in 2019^12^.

To effectively treat these common and highly resistant biofilm-related diseases, novel strategies are needed. Many laboratories are working towards such goals^13–18^. In this regard, our laboratory developed a targeted monoclonal antibody-based approach that effectively releases biofilm-resident bacteria from their protective structural matrix so that they may be more readily killed by host immune effectors and/or traditional antibiotics. To do this, we specifically focused on a ubiquitous structural constituent of the bacterial biofilm matrix, bacterial DNA-binding proteins known as the DNABII family. The two DNABII proteins, histone-like protein (HU) and integration host factor (IHF), bind to and bend double-stranded DNA^19–21^. DNABII proteins are positioned at the vertices of cross strands of extracellular DNA (eDNA) within the biofilm matrix wherein they serve as linchpin proteins that provide critical structural support to the lattice-like eDNA scaffold^22^. When biofilms formed in vitro by diverse human pathogens are incubated with antibodies directed against either a native DNABII protein or a ‘tip-chimer’ synthetic peptide immunogen we designed to mimic the immunoprotective DNA-binding ‘tips’ of a DNABII protein, the induced equilibrium shift results in rapid collapse of the biofilm with concomitant release of biofilm-resident bacteria^22–25^.

Anti-DNABII antibodies have similarly shown their disruptive potential, as well as their disease-resolution efficacy, in three pre-clinical models of human disease. Therapeutic treatment with anti-DNABII antibodies cleared aggregate *Pseudomonas aeruginosa* biofilms from the murine lung^24^ and resolved adherent mucosal biofilms formed by *Aggregatibacter actinomycetemcomitans* in a rat model of osteolytic peri-implantitis^26^. Further, when introduced into the middle ear of chinchillas, antigen-binding fragments (e.g., Fabs) derived from the humanized DNABII tip-chimer directed monoclonal mediated resolution of mucosal biofilms in a model of nontypeable *Haemophilus influenzae* (NTHI)-induced otitis media^27^. From a preventative standpoint, when used as vaccinogen, active immunization with the tip-chimer peptide induces the formation of antibodies in situ that disrupt extant biofilms formed by NTHI in the chinchilla middle ear and mediate rapid disease resolution without the need for antibiotics^25^.

Regardless of the precise method used, several laboratories report that bacteria newly released from biofilm residence exhibit a distinct, albeit transient, phenotype of increased sensitivity to antibiotic-mediated killing^18,28–33^. In addition to being more sensitive to killing than those that are biofilm-resident, as expected, one commonly observed and compelling characteristic of bacteria newly released from their biofilm fortress is that this markedly increased sensitivity to antibiotic-mediated killing is also observed in comparison to their planktonically grown counterparts, the latter of which have heretofore been considered the most antibiotic-sensitive bacterial lifestyle^23,30,31,34–36^.

Interestingly, when we evaluated susceptibility of NTHI newly released (NRel) from biofilm residence to killing by two antibiotics commonly used to treat individuals with NTHI-induced respiratory tract diseases, [e.g., amoxicillin plus clavulanic acid (A/C) or trimethoprim plus sulfamethoxazole (T/S)], NRel induced by an anti-DNABII protein monoclonal (α-DNABII) were preferentially significantly more sensitive to A/C (and not T/S) than their planktonic counterparts^36^. Further, complete proteomic analysis revealed that α-DNABII NTHI NRel exhibited a distinct proteomic expression profile compared to mid-log phase planktonically grown NTHI, as evidenced by principal component analysis^36^. Moreover, and pertinent to new work presented here, α-DNABII NTHI NRel exhibited significant > 1.5-fold increase or decrease in abundance of 103 differentially expressed proteins compared to planktonic NTHI^36^.

Here we sought to better define the kinetics of the α-DNABII NTHI NRel phenotype of preferential sensitivity to killing by a β-lactam antibiotic, specifically how quickly it develops and how long it endures. We were also interested in beginning to determine both molecular mechanisms that might underlie this sensitivity, as well as the related biological characteristics of the α-DNABII NRel population. Toward this goal, we used the well-characterized NTHI strain 86-028NP^37,38^, originally isolated from the nasopharynx of a child who underwent tympanostomy tube insertion due to chronic otitis media, as a model organism to further define and characterize the α-DNABII NRel phenotype of this predominant respiratory tract pathogen.

## Results

### Kinetic profile of antibiotic sensitivity of α-DNABII NTHI NRel to A/C or T/S

**Figure 1:**
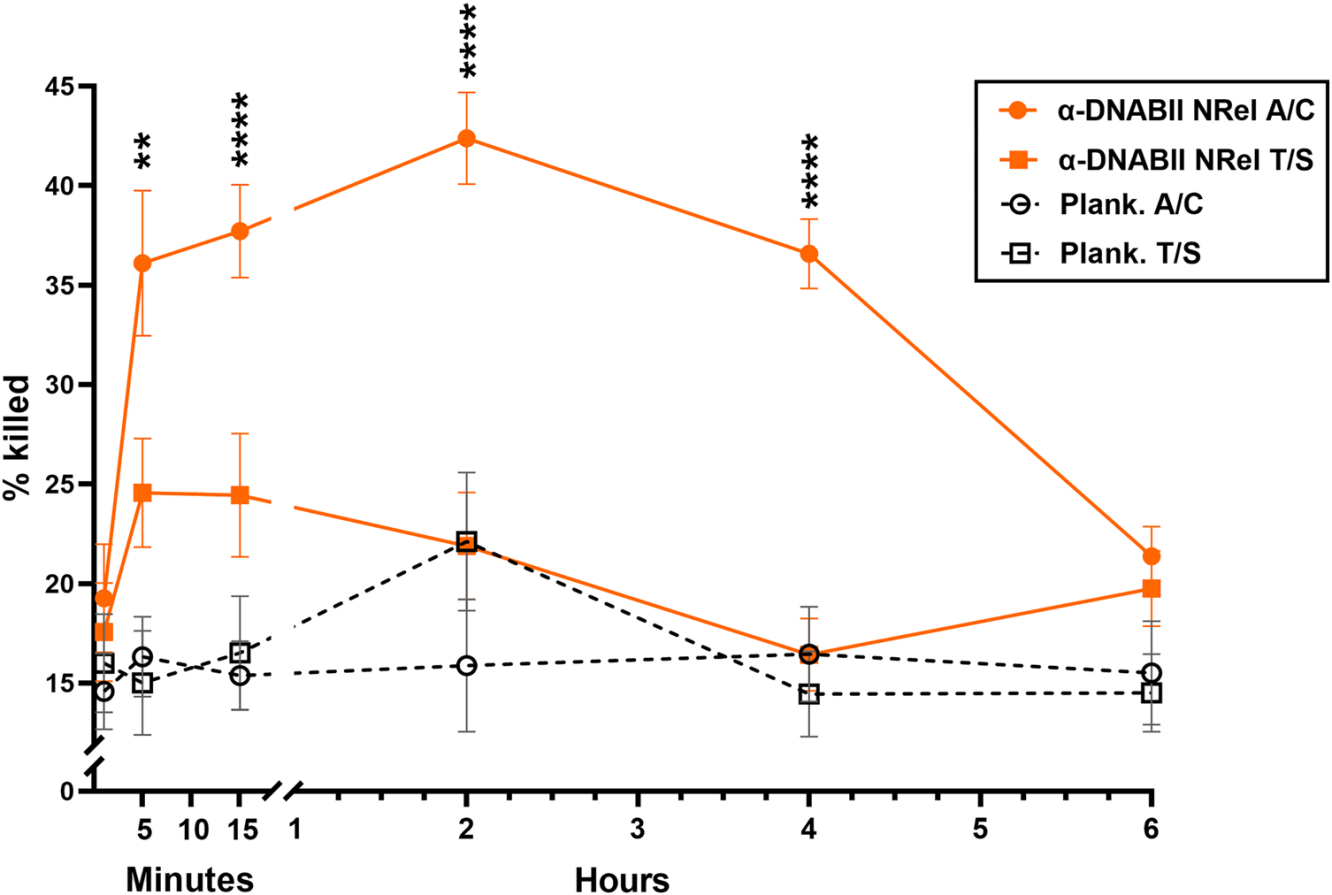
Kinetic profile of antibiotic sensitivity of α-DNABII NTHI NRel to A/C or T/S. Concentrations of antibiotics used were pre-determined to maintain killing of bacteria that grew planktonically in the fluids that overlay a biofilm in our culture system (e.g., ‘plank’ in key) to between ~ 15 and 25% to allow us to readily detect any enhanced killing of the α-DNABII NTHI NRel (e.g., ‘α-DNABII NRel’ in key). Within 5 m of biofilm exposure to α-DNABII, NTHI NRel demonstrated significantly greater killing by A/C than planktonic NTHI (*p* ≤ 0.01). By 15 m, α-DNABII NTHI NRel reached greatest level of significantly greater killing by A/C compared to that of planktonic NTHI (*p* ≤ 0.0001). Overall susceptibility to A/C peaked at 2 h of biofilm exposure to α-DNABII (*p* ≤ 0.0001) and remained significantly increased at 4 h of biofilm exposure to α-DNABII (*p* ≤ 0.0001). In contrast, T/S-mediated killing of α-DNABII NTHI NRel remained at ~ 25% or less throughout the assay period and was never statistically greater than killing of planktonic bacteria by T/S. Statistical significance was determined via two-way ANOVA with Šidák’s multiple comparisons test. **p ≤ 0.01; ****p ≤ 0.0001. Data are represented as mean ± SEM. Data shown are representative of three separate assays, each conducted on separate days, with 2–3 technical replicates per assay.

Whereas earlier work showed that NTHI NRel displayed preferential killing by a β-lactam antibiotic than by a sulfonamide after 15 m or 2 h exposure to α-DNABII^36^, here we sought to more clearly define the kinetics of this outcome. As such, after incubation of a 16 h NTHI biofilm with α-DNABII for 1 m, 5 m, 15 m, 2 h, 4 h or 6 h, we assayed killing of NTHI NRel by either A/C or T/S (Fig. 1)^39^. Antibiotic concentrations used were increased over time to adjust for bacterial growth in the culture system at the time bacteria were collected (Table 1), while simultaneously maintaining killing of NTHI that grew planktonically in fluids above the biofilms at ~ 15–25% to allow for detection of any increased sensitivity of NRel. Number (CFU/mL) of NTHI that grew planktonically in medium above the biofilm or that of NTHI released from biofilm residence by α-DNABII after the indicated period of time are listed in Supplementary Table S1. Relative ratio of recovery of α-DNABII NTHI NRel to that of those that grew planktonically in the medium above the biofilm ranged from 1.3:1 to 2.4:1, similar to what we previously reported^40^.

**Table 1:** Concentrations of A/C or T/S used to determine the kinetic profile of antibiotic sensitivity of α-DNABII NTHI NRel when recovered at increasing time points within the assay period. Antibiotic concentrations are listed as a ratio of amoxicillin trihydrate (µg/mL) to clavulanic acid (µg/mL) (A/C), or of trimethoprim (µg/mL) to sulfamethoxazole (µg/mL) (T/S). We increased the antibiotic concentrations used after 2 h to account for bacterial growth in the culture system while also maintaining killing of NTHI that grew planktonically within the fluids that overlay a biofilm in our negative control culture systems between ~ 15 to 25% at any given timepoint.

Timepoint	A/C concentration	T/S concentration
1 m	0.8/1.0	0.2/1.0
5 m	0.8/1.0	0.2/1.0
15 m	0.8/1.0	0.2/1.0
2 h	2.5/1.3	1.0/5.0
4 h	2.5/1.3	1.0/5.0
6 h	2.5/1.3	1.0/5.0

At every time point tested, T/S-mediated killing of α-DNABII NTHI NRel never exceeded 25%, consistent with our previous report of minimal relative susceptibility to this antibiotic when tested on α-DNABII NRel recovered at 15 m or 2h^36^. Killing of α-DNABII NTHI NRel by T/S was greatest at 5 and 15 m but declined by 2 h and remained at or below 20% at both 4 h and 6 h. Killing of α-DNABII NTHI NRel by T/S was never significantly greater than that of planktonic NTHI by T/S.

Conversely, sensitivity of α-DNABII NTHI NRel to killing by A/C was significantly greater than that of planktonic NTHI within 5 m (*p* = 0.003). This preferential sensitivity to the β-lactam antibiotic peaked within NRel recovered after exposure of the biofilm to α-DNABII for 2 h (*p* = 0.0001), a degree of significant difference that was maintained at the 4 h timepoint (*p* < 0.0001). By 6 h, the susceptibility of α-DNABII NTHI NRel to killing by A/C was no different than that of planktonic killing by A/C or T/S.

Given that NTHI NRel recovered at the 2 h timepoint were significantly sensitive to killing by A/C, whereas those recovered at the 6 h timepoint had lost this specific characteristic, we next focused on comparative evaluation of these two NRel populations to begin to determine what might contribute to the noted enhanced A/C sensitivity.

### 2 h and 6 h α-DNABII NTHI NRel were distinct in relative transcription of a panel of genes whose products were likely involved in sensitivity to a β-lactam antibiotic

To begin to determine the relative differences in sensitivity to killing by A/C between 2 h α-DNABII NTHI NRel (‘2 h NRel’) and those recovered at 6 h (‘6 h NRel’), which no longer displayed this characteristic, we established a set of 15 primer pairs (Supplementary Table S2) designed to specifically characterize sensitivity to a β-lactam antibiotic via real-time quantitative reverse transcription PCR (qRT-PCR).

**Figure 2:**
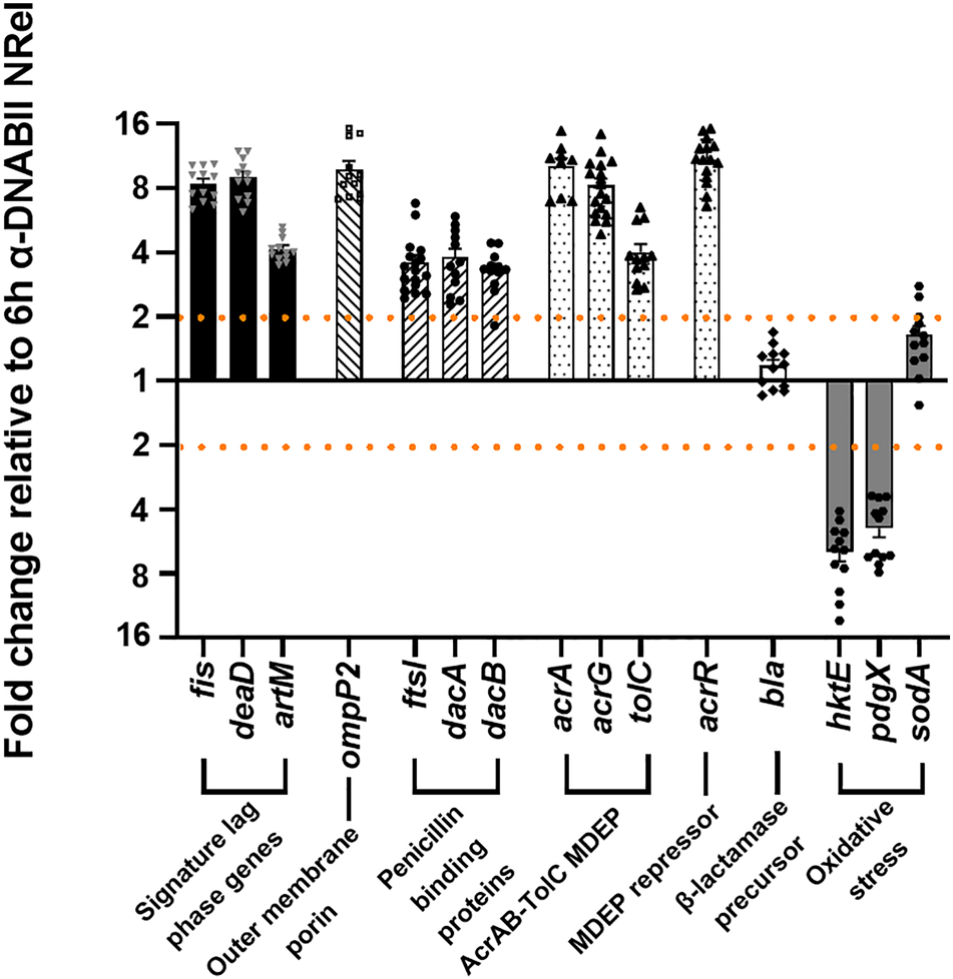
2 h and 6 h α-DNABII NTHI NRel were distinct in relative transcription of a panel of genes whose products are known to be involved in sensitivity to a β-lactam antibiotic. By qRT-PCR, 2 h and 6 h α-DNABII NTHI NRel were transcriptionally distinct via analysis of 15 targeted profiled genes. 11 of the 15 genes profiled were significantly (> twofold change) up-regulated and two genes were significantly down-regulated in 2 h α-DNABII NTHI NRel compared to NRel collected after 6 h. Data are represented as mean ± SEM. Analysis of each gene occurred at least three times on separate days with 2–3 technical triplicates per assay. [Note: MDEP – multidrug efflux pump].

The first subset profiled are three canonical lag phase genes (*fis*, *deaD* and *artM*), used here as in earlier work where we showed that NTHI NRel recovered after 15 m of biofilm exposure to α-DNABII exhibited both an abundance of ribosomal proteins characteristic of bacteria in lag phase and significantly (*p* ≤ 0.05) greater transcript abundance of these three genes compared to planktonic NTHI^36,41^. Thus, we now wondered if the 2 h NRel, which showed preferential sensitivity to A/C, were likely still in lag phase but were no longer so at the 6 h time point. These three canonical lag phase genes did indeed exhibit a significant approximately 4- to eightfold greater abundance at 2 h compared to 6 h (Fig. 2). Thereby, these data provided further evidence that upon release from biofilm residence by α-DNABII, NTHI NRel appeared to mimic bacteria in lag phase within 15 min and up until at least 2 h after release.

We next assessed relative expression of *ompP2*, which encodes OMP P2, the major outer membrane porin of NTHI^42,43^, as this porin is likely involved in access of A/C into the bacterial cell. Relative to 6 h NRel, 2 h NRel exhibited a significant nearly tenfold increased expression of *ompP2*. Thus, this finding also provided a potential mechanism for the observed significantly greater killing of 2 h NRel by A/C, which was no longer observed at 6 h.

The next set of three genes (e.g., *ftsl*, *dacA*, and *dacB*) encode bacterial penicillin binding proteins (PBPs), which are targets of β-lactam antibiotics^44–48^. We found a significant threefold increase in expression of each PBP gene in 2 h NRel compared to 6 h NRel, a finding which again suggested a potential mechanism for the observed increased sensitivity to A/C that was detectable shortly after release from biofilm residence by α-DNABII.

Next, we evaluated relative expression of three AcrAB-TolC multidrug efflux pump (MDEP) genes due to their role in removal of antibiotics from the bacterial cell^49^. We found significantly increased expression (≥ fourfold) of each of these three genes in 2 h vs. 6 h NRel, which suggested greater activity of this efflux pump at the 2 h time point. Interestingly, however, the gene that encodes for the repressor of this efflux pump, *acrR*, was also significantly up-regulated (11-fold) in 2 h NRel. Given the paradoxical nature of these findings with regard to the significant A/C sensitivity, we considered whether collection of RNA at the exact time point at which the biological activity of relative antibiotic killing was assessed (e.g., 2 or 6 h) might have provided the wrong ‘snapshot in time’ to gauge the actual activity of these particular gene products.

As such, we also recovered RNA after 30 m of biofilm exposure to α-DNABII (Supplemental Fig. S1) as perhaps a better surrogate for comparative gene product activity. We found significantly increased transcript abundance for *acrA*, *acrG*, and *tolC* at 30 m relative to 2 h (*p* = 0.004 for *acrA* or *p* = 0.0005 for *acrG* and *tolC*). Additionally, there was significantly greater *acrR* transcript abundance at 30 m compared to at 2 h (*p* ≤ 0.0001). Unfortunately, these additional data did not offer clarity as to the role of this efflux pump in the observed enhanced sensitivity of α-DNABII NRel in this strain of NTHI to A/C, but suggested that overall the net relative biological outcome of these four gene products favored activity of the repressor, as others have similarly observed^30^. However, it is also possible that the role of AcrR is not clear in this strain. Further, it is important to note that time sensitivity of gene expression changes and how that might relate to relative protein expression and/or the stability or lifespan of these gene products (as well as likely others) potentially played additional roles here.

Whereas we used amoxicillin with the β-lactamase inhibitor clavulanic acid, given the observed significant sensitivity to killing by A/C in 2 h NRel, we were interested in relative expression of *bla,* which encodes the NTHI β-lactamase precursor. We found no significant difference between the 2 h and 6 h NRel, which suggested that this gene product likely played no role in the transient significant sensitivity to A/C observed.

Lastly, we profiled three genes involved in bacterial resistance to oxidative stress, *hktE*, *pdgX* and *sodA*, as given the previously demonstrated rapid efficacy of α-DNABII when used therapeutically in three pre-clinical models of human disease without addition of antibiotics^24,26,27^, we were curious about potential vulnerability of NTHI NRel to host innate immune effectors in addition to antibiotics. Whereas there was no significant difference in relative *sodA* gene expression, *hktE* and *pdgX* were significantly down-regulated by ≥ fourfold in 2 h NRel compared to 6 h NRel. This outcome suggested that the α-DNABII NTHI NRel phenotype might also include being less well equipped to mitigate oxidative stress. Thereby, we now wanted to determine the relative sensitivity of 2 h *vs* 6 h NRel to killing by human PMNs.

### 2 h α-DNABII NTHI NRel were significantly more susceptible to NADPH-oxidase sensitive intracellular killing by human PMNs

**Figure 3:**
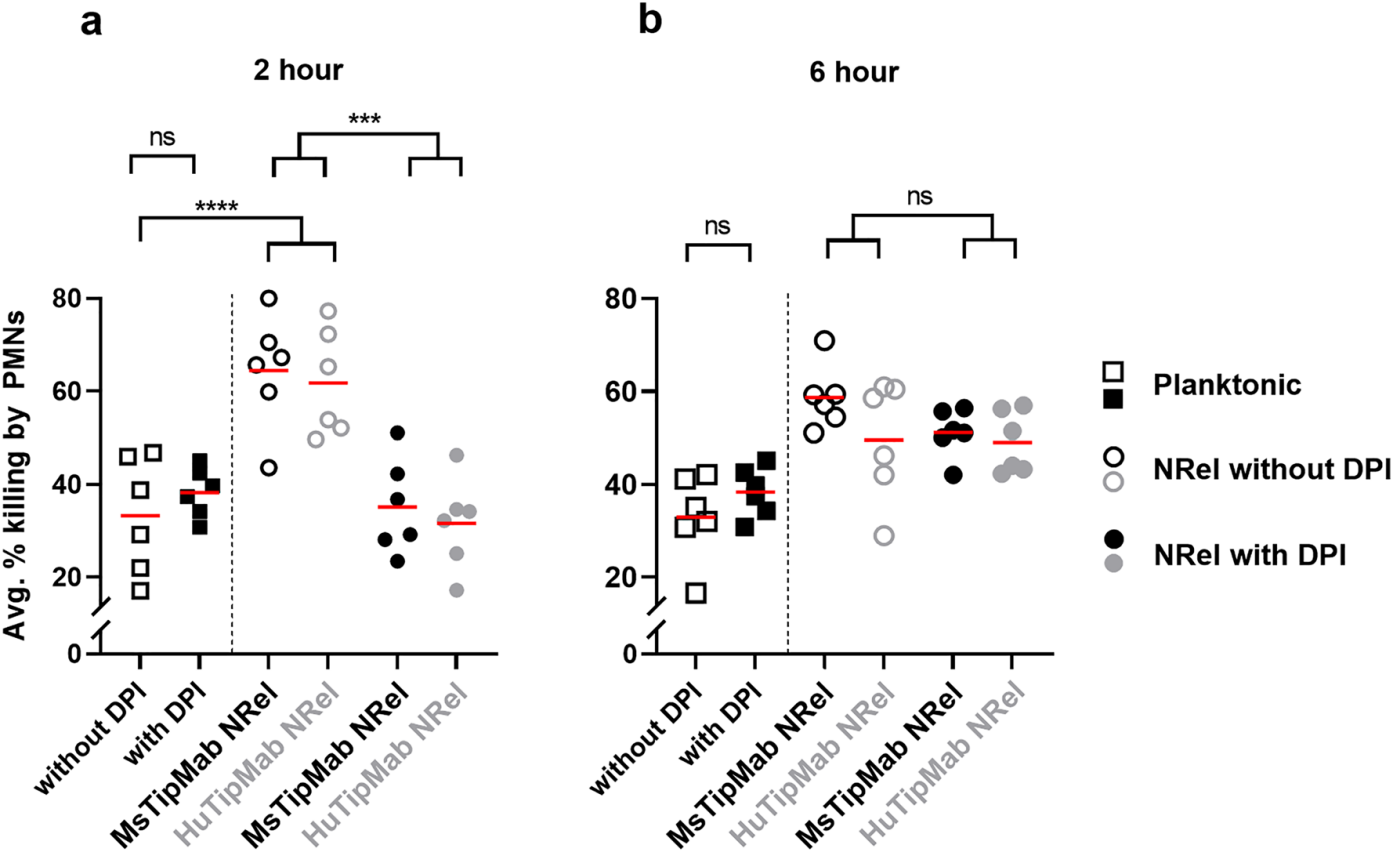
2 h α-DNABII NTHI NRel were significantly susceptible to NADPH-oxidase sensitive intracellular killing by human PMNs. Here we determined the relative ability of activated human PMNs to kill isogenic planktonic, 2 h or 6 h α-DNABII NTHI NRel. (**A**) At the 2 h time point, ~ 33% of planktonic NTHI were killed by activated human PMNs. However, regardless of whether induced by MsTipMab or HuTipMab, NTHI NRel were significantly (*p* ≤ 0.0001) more susceptible to killing by human PMNs as evidenced by ~ 65% and ~ 62% killing, respectively. DPI treatment of human PMNs significantly reduced susceptibility of 2 h NTHI NRel whether induced by MsTipMab or HuTipMab, as evidenced by killing at ~ 35% or ~ 32% respectively (*p* ≤ 0.001). (**B**) By 6 h, use of DPI to inhibit intracellular NADPH-oxidase did not affect the relative ability of activated human PMNs to kill either the MsTipMab- or HuTipMab-induced NRel. There was no difference in susceptibility to killing by DPI-treated PMNs for planktonic NTHI relative to 2 h NRel or relative to 6 h NRel (*p* > 0.05). Red lines indicate the mean. Statistical significance comparing planktonic NTHI killed by untreated or DPI-treated PMNs was determined via unpaired two-tailed t-test or two-way ANOVA with Šidák’s correction (Panels **A-B**). ***p ≤ 0.001; ****p ≤ 0.0001. Data presented are mean percent killings from at least six separate assays, each conducted on separate days with two–three technical replicates per data point.

To assess the relative ability of activated human PMNs to kill 2 h *vs* 6 h NRel, we introduced use of the humanized version of our anti-tip chimer monoclonal antibody (‘HuTipMab’)^50^, while continuing to use the murine monoclonal (α-DNABII) we had used to this point but will now refer to as ‘MsTipMab’ for clarity. This direct comparison of HuTipMab to MsTipMab allowed us to expand our evaluation as to whether humanization compromised any assessed activity of this monoclonal, including induction of the NRel phenotype. We found that whereas susceptibility of planktonic NTHI to killing by human PMNs was ~ 33% (Fig. 3A, open box symbols), that of 2 h NTHI NRel, whether induced by either Ms- or HuTipMab, was significantly greater (65% or 62%, respectively, *p* ≤ 0.0001) (Fig. 3A).

To next determine if the enhanced killing of 2 h NRel by human PMNs was likely a result of their limited relative ability to mitigate oxidative stress (due to significant down-regulation of *hktE* and *pdgX*), we incorporated the intracellular NADPH-oxidase inhibitor diphenyleneiodonium chloride (DPI)^51^ into our PMN killing assay. When neutrophils were incubated with DPI, 2 h NRel induced by Ms- or HuTipMab were now significantly less susceptible to killing (35% and 32%, respectively, *p* = 0.0006) compared to killing by untreated PMNs (Fig. 3A , closed black and grey circles). Notably, regardless of whether induced by incubation with Ms- or HuTipMab, killing of 2 h NRel by DPI-treated PMNs was now no longer statistically significantly different than that of planktonic NTHI by pre-treated PMNs (*p* = 0.36). DPI had no effect on killing of planktonic.

In 6 h NRel, DPI treatment had no effect on the relative ability of human PMNs to kill these bacteria once phagocytized, as there was no significant difference in the susceptibility to killing by DPI-treated PMNs compared to untreated PMNs (*p* = 0.3) (Fig. 3B). As observed in assay of 2 h NRel, there was no difference in susceptibility to killing by DPI-treated PMNs of planktonic NTHI relative to 6 h NRel (*p* = 0.10), and killing of planktonic NTHI appeared largely unaffected by the addition of DPI, which suggested a greater influence of extracellular mechanisms of PMN-mediated killing for planktonic NTHI. Moreover, there was no difference in susceptibility to killing by DPI-treated PMNs in 2 h relative to 6 h NRel (*p* = 0.07).

Taken together, these data indicated that in addition to the transient, yet notably increased sensitivity to killing by A/C, α-DNABII NTHI NRel were also highly vulnerable to intracellular killing by activated human PMNs, and specifically in an NADPH-oxidase sensitive manner. This characteristic was no longer detected in 6 h NRel which suggested yet another transient vulnerability of this phenotype.

### Bacterial membrane permeability was significantly greater in 2 h vs 6 h α-DNABII NTHI NRel

Given that *ompP2* was significantly up-regulated in 2 h NRel compared to 6 h NRel, we wondered if this increased expression of the major NTHI outer membrane porin might correlate with greater membrane permeability. To address this, we used the nucleic acid intercalating green fluorescing dye, SYTOX Green. SYTOX Green can only access the NTHI genome if both the inner and outer membranes of this Gram-negative bacterium are permeable, thereby increased fluorescence signal was used as a surrogate of relative outer membrane permeability.

**Figure 4:**
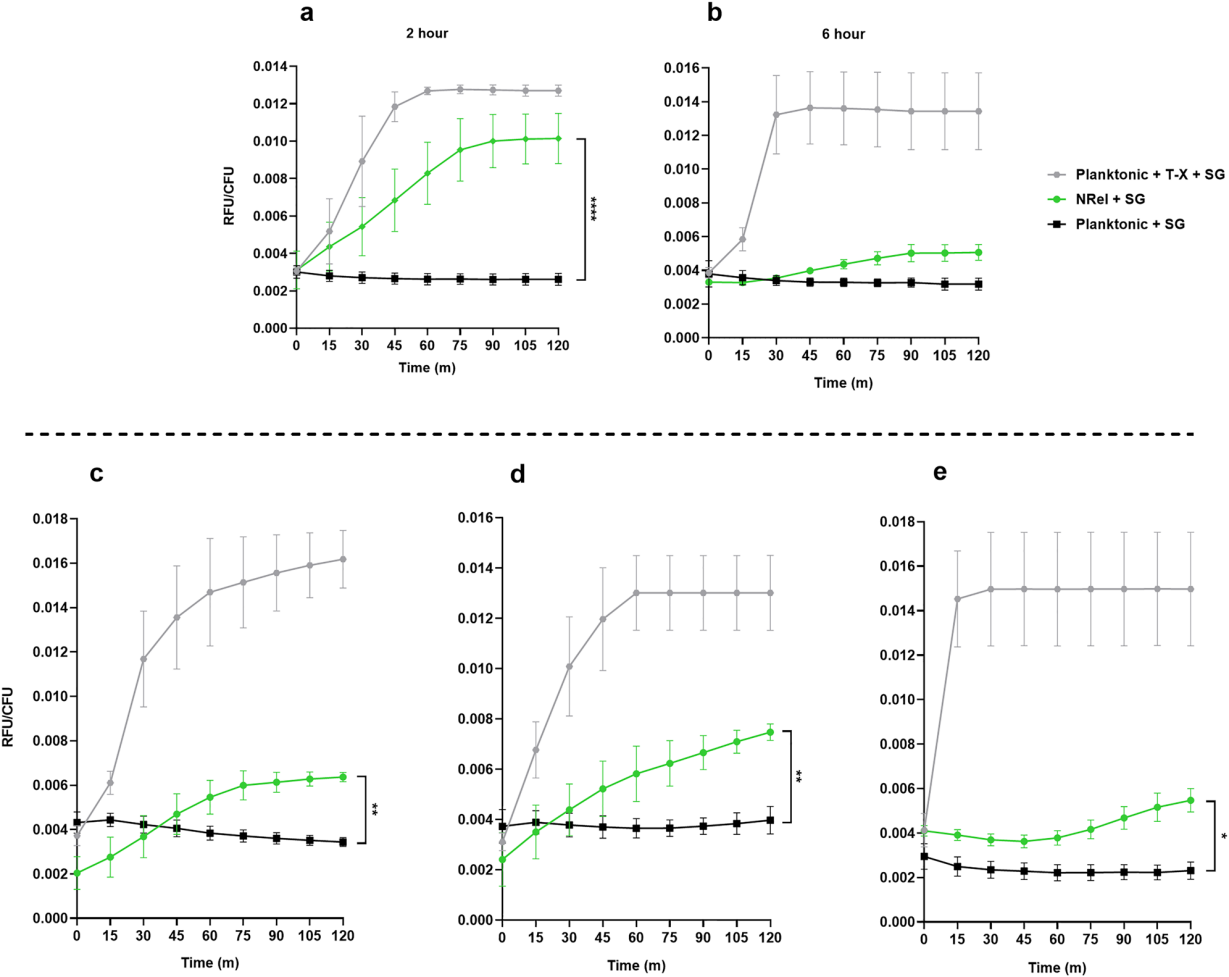
Bacterial membrane permeability was significantly greater in 2 h α-DNABII NRel compared to those that were planktonically grown for NTHI plus three additional human pathogens. Relative emitted fluorescence was measured by the nucleic acid intercalating dye SYTOX Green as a proxy for assessing outer membrane permeability of α-DNABII NRel. In each assay, planktonically grown bacteria with or without treatment with Triton X-100 served as the positive and negative controls, respectively. (**A**) 2 h α-DNABII NTHI NRel were significantly (*p* ≤ 0.0001) more fluorescent over the 120 m assay time period than the negative control. (**B**) By 6 h, the exhibited fluorescence of α-DNABII NTHI NRel resembled that of planktonically grown NTHI. The observed increased membrane permeability of the 2 h but not 6 h α-DNABII NTHI NRel supported the observed transient and significantly greater sensitivity of 2 h NRel to A/C that was no longer observed in the 6 h NRel population. (**C-E**) To determine if increased outer membrane permeability might be a more universal characteristic of α-DNABII NRel, we also assessed membrane permeability of identically generated 2 h α-DNABII NRel of two additional Gram-negative pathogens and one Gram-positive pathogen. α-DNABII *E. coli* NRel (Panel **C**) exhibited significantly increased (*p* ≤ 0.01) fluorescence compared to the negative control, as did α-DNABII *P. aeruginosa* NRel (Panel **D**) (*p* ≤ 0.01) and MRSA NRel (Panel **E**) (*p* ≤ 0.05). Data are presented as mean ± SEM. Data represent three separate assays conducted on three separate days with three technical triplicates per assay. Relative fluorescence units (RFU) were normalized to CFU of respective bacterial population added to the assay plate. Statistical analyses of respective triplicate runs were calculated via repeated measures linear mixed model for comparison of time course data between values of NRel versus those of the negative control.

In an assay of both the 2 h and 6 h NRel, the negative control (mid-log phase planktonically grown NTHI) was minimally fluorescent immediately after exposure to SYTOX Green, then maintained this low level of fluorescence throughout the 120 m assay period (Fig. 4A and B, black lines). Conversely, the positive control (mid-log phase planktonically grown NTHI treated with Triton X-100 to artificially permeabilize the bacterial membrane) also demonstrated a low level of fluorescence at time zero, but then rapidly increased to reach and maintain maximum fluorescence of ~ 0.0125 RFU/CFU within approximately 30–45 m (Fig. 4A and B, grey lines). The 2 h NRel also demonstrated a low level of fluorescence immediately upon exposure to SYTOX Green, but then steadily increased over time to reach a maximum plateau at 75 m which approached that of the positive control (Fig. 4A, green line). Throughout the assay, 2 h NRel consistently fluoresced significantly greater than planktonically grown NTHI (*p* ≤ 0.0001), which suggested increased outer membrane permeability in 2 h NRel.

Given that 6 h NRel demonstrated limited sensitivity to either A/C or T/S (see Fig. 1), these 6 h NRel were also assayed to determine their relative outer membrane permeability (Fig. 4B). Whereas planktonic NTHI (with and without Triton X-100 treatment) behaved as described in Fig. 4A, 6 h NRel now emitted a fluorescence signal only slightly greater than mid-log phase planktonically grown NTHI throughout the 120 m assay period (*p* = 0.11). These data suggested that in alignment with enhanced A/C sensitivity, increased outer membrane permeability observed in 2 h NRel was also transient and had resolved by 6 h.

To ensure that the observed increased outer membrane permeability was not an NRel characteristic exclusive to either NTHI as a whole, or to this particular NTHI isolate, we also assessed identically generated NRel of three additional pathogens [*Escherichia coli*, *Pseudomonas aeruginosa*, or a methicillin-resistant *Staphylococcus aureus* (MRSA)] as induced upon exposure of respective 16 h biofilms to α-DNABII for 2 h. Whereas each bacterium demonstrated a unique pattern, 2 h α-DNABII NRel of *E. coli*, *P. aeruginosa* and MRSA all exhibited significantly increased fluorescence signal (*p* = 0.007 for *E. coli* NRel, *p* = 0.005 for *P. aeruginosa* NRel, *p* = 0.02 for MRSA NRel) compared to their planktonically grown counterparts over the 120 m assay period (Fig. 4C-E). These data suggested that a transient phenotype of increased outer membrane permeability was potentially a common characteristic of bacteria released from biofilm residence by the action of a monoclonal antibody directed against the DNABII structural biofilm matrix proteins.

## Discussion

The need for more effective therapeutic approaches, or preferably prevention strategies, to combat persistent biofilm-related diseases cannot be overstated. Biofilm-related diseases exacerbate the global major public health crisis of antibiotic resistance, as despite the fact that biofilm-resident bacteria are significantly recalcitrant to antibiotic treatment, individuals with these diseases are nonetheless commonly prescribed antibiotics as one of our very limited repertoire of treatment options^52–54^. Not only are bacteria resident within biofilms highly resilient to antibiotics, but resistance of biofilms to clearance by immune effectors adds complexity by significantly challenging the host’s ability to resolve biofilm-related diseases^55–57^. To address these issues, one such strategy includes the use of methodologies that can release biofilm-resident bacteria from their protective fortress to facilitate their killing by the host and, if needed, by co-delivery of traditional antibiotics that may now be substantially more effective.

Towards this goal, we’ve focused on a key structural component of the bacterial biofilm matrix, the bacterial DNA-binding proteins of the DNABII family^19–21^. The two DNABII proteins, HU and IHF, bind to crossed-strands of extracellular DNA in the biofilm matrix, thereby effectively stabilizing this structure^22^. When we incubate a biofilm formed either individually, or by two of any of six diverse respiratory tract pathogens, as well as any of the highly antibiotic resistant members of the ESKAPEE pathogens, with a monoclonal antibody directed against the protective DNA-binding ‘tips’ of a DNABII protein, the biofilm rapidly collapses^22–25,40^. This collapse releases all tested biofilm-resident pathogens into a highly vulnerable, but transient, state wherein they are now significantly more susceptible to commonly used antibiotics of multiple classes in vitro, and also to innate immune effectors *in vivo*^24–27,35,36,58,59^. This enhanced susceptibility is observed not only when compared to their isogenic counterparts resident in the biofilm state, but more importantly, is also greater than those in the planktonic state.

Specifically, in an earlier report, we showed that after incubation of a biofilm formed by a clinical isolate of NTHI with α-DNABII for either 15 min or 2 h, the resultant NRel were significantly more susceptible to killing by A/C than they were to killing by T/S^36^. Overall, that the NRel were more sensitive to killing than were their biofilm-resident counterparts was not unexpected. However, that these NRel were just as or significantly more sensitive to either antibiotic than their isogenic planktonically grown counterparts, was indeed notable^36^. Here, we investigated more thoroughly the observed preferential greater sensitivity of NRel to killing by the β-lactam antibiotic A/C, than by the sulfonamide, T/S.

Collectively, data presented here revealed more clearly the kinetics of the A/C-sensitive α-DNABII NRel phenotype after release from residence in a biofilm formed by NTHI strain 86-028NP by a murine monoclonal directed against a biofilm structural matrix protein of the DNABII family. We found that this significant sensitivity is detectable within 5 min and endures for approximately 6 h in vitro*,* after which the NRel population is no longer selectively more sensitive to A/C.

Examination of relative expression of several genes whose products are known to contribute to β-lactam sensitivity revealed multiple factors that likely contributed to the increased susceptibility of α-DNABII NTHI NRel to A/C. In earlier work, we showed that within 15 min of release from biofilm residence by anti-DNABII, NTHI exhibit increased expression of genes characteristic of bacteria in lag phase, a period during which bacteria are involved in repair of their cell envelopes and membranes, and thereby are also more membrane permeable^41,60^. Here, we confirmed that at the 2-h time point, α-DNABII NTHI NRel still appeared to mimic bacteria in lag phase as evidenced by significant up-regulation of the same three canonical lag phase genes compared to NRel recovered at the 6 h time point. That α-DNABII NTHI NRel never displayed significantly greater susceptibility to T/S was likely explained by the understanding that bacteria in lag phase are not highly active in protein synthesis (a target for the sulfonamide class of antibiotics, like T/S)^61–63^.

In previous data from our group^36^ complete proteomic analysis of 15 min α-DNABII NRel showed an increase in the peptidoglycan synthesis protein, MurB, which suggests altered cell envelope composition^36,64^. Modification in the α-DNABII NTHI NRel cell envelope was further corroborated by new results shown here of increased transcript abundance of *ompP2*, which encodes for the major porin of the NTHI outer membrane, thus providing a mechanism for antibiotic entry into the bacterial cell. Moreover, this increased expression of *ompP2* aligned with our concomitant demonstration of a similarly timed transient increase in outer membrane permeability in newly released NTHI at the 2-h time point, as evidenced by relative fluorescence upon incubation with SYTOX Green.

New data presented here also demonstrated that significant increases in relative expression of three profiled PBPs is expected to have also contributed to the heightened antibiotic sensitivity shown by α-DNABII NTHI NRel, as β-lactams target PBPs^44–46^. Indeed, specific to relative increased expression of the *H. influenzae ftsI* gene, bacteria that have evolved mutations in the transpeptidase region of this encoded protein demonstrate increased β-lactam resistance^44–48^. Lastly, 2-h α-DNABII NTHI NRel showed limited expression of *bla*, the β-lactamase precursor, which suggested yet another potential contributor to the significant sensitivity to killing by A/C, as even in the absence of clavulanic acid, NTHI NRel would have limited ability to degrade this antibiotic once it had entered the cell^65^. That NTHI newly released from their protective biofilm fortress seem to mimic bacteria in lag phase and exhibited significant up- or downregulation of numerous profiled genes may be explained by the fact that bacteria resident within a biofilm are often metabolically quiescent^8^. As such, when rapidly released from biofilm residence by the action of anti-DNABII, we posit that bacteria are released into a state wherein they are transiently ill equipped to mediate the killing functions of antibiotics and human PMNs. Whereas these defensive functions are ultimately regained, the NRel phenotype nonetheless provides a window of opportunity for more effective eradication of the formerly biofilm-resident bacteria.

Whereas increased susceptibility to antibiotics has heretofore been reported as a characteristic of the newly released phenotype for multiple bacteria^23,30,31,34–36^, we were also interested in exploring whether there also might be evidence of increased susceptibility to immune effectors. We were specifically interested in susceptibility to innate immune effectors as to date, and in three separate pre-clinical models of human disease, we have demonstrated that when newly released from a biofilm by the action of a DNABII-directed antibody, bacteria and any biofilm remnants are rapidly cleared by the respective host in the absence of any added antibiotic. We reported this for mucosal biofilms formed in the middle ear by NTHI in a chinchilla model of experimental otitis media, for aggregate biofilms formed in the murine lung by *Pseudomonas aeruginosa* and for biofilms formed in the oral cavity by *Aggregatibacter actinomycetemcomitans* in a rat model of periimplantitis^24–27^. This antibiotic-free efficient bacterial clearance and rapid disease resolution suggested to us that innate immune effectors, particularly PMNs, were likely involved. Our interest was further piqued when here we showed that α-DNABII NTHI NRel demonstrated transient marked down-regulation of two enzymes (a catalase and a peroxiredoxin-glutaredoxin) important to mitigation of oxidative stress^66–69^.

As such, we assessed the relative killing of α-DNABII NTHI NRel by human PMNs given their role as a first line of defense against unwelcome pathogens. PMNs elicited to the site of infection exhibit antimicrobial activities in a variety of ways. Chief among their arsenal is extracellular killing via NETosis, as well as intracellular killing by reactive oxygen species after phagocytosis^70,71^. We found that NTHI newly released from biofilm residence by α-DNABII were indeed transiently, yet highly vulnerable to killing by activated human PMNs. While this killing was overall likely due to both extra- and intracellular means, there was significant reduction in killing when PMNs were pre-treated with the specific intracellular NADPH-oxidase inhibitor, DPI^51^. This finding aligned well with the similarly transient marked reduced expression of both *hktE* and *pdgX* by the NRel population. Use of DPI also allowed us to mimic the limited functionality of PMNs recovered from individuals with chronic granulomatous disease who also exhibit increased vulnerability to fungal and bacterial infections, including those induced by pathogens capable of forming highly recalcitrant biofilms^51,72^. Finally, it is worth noting that there was no difference in heightened susceptibility to killing by PMNs whether NTHI were released from biofilm residence by the action of either the murine or humanized α-DNABII monoclonal. Thus, these data add to others which show that the humanization process did not diminish the activity of this monoclonal antibody in any way tested to date^27,35^.

To determine if specific NRel attributes were perhaps more broadly demonstrable in bacteria other than NTHI, we also explored the characteristic of increased outer membrane permeability for three additional human pathogens released from biofilm residence by action of the humanized monoclonal directed against a DNABII protein. This particular characteristic was indeed shared by α-DNABII NRel of two additional Gram-negative pathogens, *E. coli* and *P. aeruginosa,* as well as by an isolate of the Gram-positive pathogen methicillin-resistant *S. aureus*, which suggested that transient increased outer membrane permeability might be a common characteristic of the α-DNABII NRel phenotype. A potential limitation of this study is that we cannot guarantee that α-DNABII NRel were exclusively comprised of only those bacteria newly released from biofilm residence, as they may also have included bacteria that left biofilm residence as part of natural biofilm remodeling^73^. Nonetheless, this possibility did not limit our ability to observe and describe distinct phenotypes of those bacteria newly released from biofilm residence compared to their planktonically grown counterparts.

Collectively, new data provided here support continued validation of a therapeutic approach wherein we propose to deliver the humanized monoclonal antibody to an individual with a recalcitrant biofilm infection to enable the host’s innate immune system to effectively eradicate those bacteria newly released from the biofilm, ideally in a controlled manner. To date, three separate pre-clinical models of disease support this antibiotic-free strategy^24–26^. However, if needed or warranted, and given that significant antibiotic sensitivity was realized within minutes, we would propose a combinatorial approach wherein a now effective antibiotic is co-delivered to promote rapid killing of the bacteria as they are released from the pathogenic biofilm. In a highly antibiotic resistant world where bacterial biofilms are persistent and recalcitrant to both conventional antibiotic treatment and host immune clearance, this pathogen-agnostic anti-DNABII approach may provide a powerful and broadly effective novel strategy that offers promising therapeutic potential in the absence of new antibiotic development for eradication of biofilm-related diseases.

## Materials and methods

### Ethics statement

De-identified human blood donations provided by healthy adult subjects that span the demographic spectrum of central Ohio were made under the auspices of the Research Institute Blood Donor Services of Nationwide Children’s Hospital after informed written consent was obtained. PMNs were isolated from these blood specimens for use in studies conducted within our laboratory in conformity with, and as approved under, Nationwide Children’s Hospital Institutional Biosafety Committee (IBC) protocol #IBS-00000449. All experiments were conducted in accordance with relevant guidelines and regulations of the Nationwide Children’s Hospital Research Institute Blood Donor Services and Institutional Biosafety Committee.

### Bacterial strain and growth

Nontypeable *Haemophilus influenzae* (NTHI) strain 86-028NP^37,38^ was maintained frozen in LN_2_ at passage #4 on artificial medium since its original isolation from the nasopharynx of a child who underwent tympanostomy tube insertion due to chronic otitis media. NTHI 86-028NP was grown in Brain Heart Infusion broth supplemented (sBHI) with hemin (2 µg/mL) (Sigma-Aldrich, Cat no. H9039) and β-NAD (2 µg/mL) (Sigma-Aldrich, Cat no. N1511) at 37 °C with 5% CO_2_ in a humidified atmosphere. Methicillin-resistant *Staphylococcus aureus* (MRSA) (clinical isolate from child with cystic fibrosis) or *Pseudomonas aeruginosa* strain 142-1^35^ (University of North Texas) were grown on Tryptic Soy agar (TSA) or in Tryptic Soy broth. *Escherichia coli* strain UTI89^22^ was grown on Lysogeny Broth agar or in Lysogeny broth (LB) for 18–24 h at 37 °C, 5% CO_2_ in a humidified atmosphere.

### Antibodies

A murine (Rockland Immunochemicals, Inc., Philadelphia, PA)^24^ or humanized (Lake Pharma, Inc., San Carlos, CA)^35,50^ monoclonal antibody of the IgG isotype against a tip-chimer peptide designed to mimic protective epitopes of the DNA-binding ‘tips’ of the alpha and beta subunits of a bacterial DNABII protein were prepared for us under contract to Rockland Immunochemicals, Inc. or Lake Pharma, Inc., respectively. These monoclonal antibodies are referred to as MsTipMab (murine) or HuTipMab (humanized).

### Collection and quantitation of α-DNABII NTHI NRel

Two and a half mL NTHI, *E. coli* UTI89, *P. aeruginosa* 142-1 or MRSA at 2 × 10^5^ CFU/mL were seeded into separate 10 cm^2^ flat tissue culture tubes (TPP, Trasadingen, Switzerland, Cat no. 91243) and allowed to establish when incubated statically at 37 °C with 5% CO_2_ in a humidified atmosphere in respective medium for 16 h. After 16 h, tubes were carefully inverted in the incubator. Medium containing non-adherent bacteria was poured off. While inverted, 2.5 mL equilibrated (37 °C, 5% CO_2_) Dulbecco’s phosphate buffered saline without calcium or magnesium (DPBS) was added. The tubes were rotated 360° to gently remove additional non-adherent bacteria, and the DPBS was poured off as above. To generate α-DNABII NTHI NRel, washed biofilms were incubated at 37 °C with 5% CO_2_ in a humidified atmosphere for 1 m, 5 m, 15 m, 2 h, 4 h, or 6 h with either MsTipMab or HuTipMab at a concentration of 5 µg antibody diluted in sBHI/0.8 cm^2^. Number of NTHI released from biofilm residence by α-DNABII or those growing in the fluids above the biofilm at a given timepoint is supplied in Supplementary Table S1. To yield α-DNABII *E. coli* UTI89, *P. aeruginosa* 142-1 or MRSA NRel, respective 16 h biofilms were incubated at 37 °C, 5% CO_2_ in a humidified atmosphere for 2 h with HuTipMab at a concentration of 5 µg antibody diluted in sBHI/0.8 cm^2^.

### Antibiotic sensitivity of α-DNABII NTHI NRel by A/C or T/S

To determine the kinetics of the relative sensitivity of the α-DNABII NTHI NRel to antibiotic-mediated killing, we incubated the NRel with either amoxicillin (Sigma-Aldrich, Cat no. 31586) and clavulanate lithium (Sigma-Aldrich, Cat no. 1134426) (A/C) or with trimethoprim (Sigma-Aldrich, Cat no. T7883) and sulfamethoxazole (Sigma-Aldrich, Cat no. 723-46-6) (T/S) as previously described, with modifications^36^. As above, 16 h NTHI biofilms were established, washed, and treated in tissue culture tubes for 1 m, 5 m, 15 m, 2 h, 4 h, or 6 h to yield α-DNABII NTHI NRel of different ‘ages’. α-DNABII NTHI NRel were then carefully poured into Eppendorf tubes and sonicated for 2 m in a water bath sonicator to disperse bacterial aggregates. 90 µL aliquots of the bacterial suspensions were added to a 96-well plate, followed by 10 µL of either A/C or T/S. We pre-determined the antibiotic concentration that would maintain killing of NTHI that reside in the fluids that overlay a biofilm in our culture system between ~ 15 and 25% to allow us to readily detect and quantify any enhanced killing of the α-DNABII NTHI NRel^36^. Concentrations of antibiotics used at each time point are listed in Table 1. As a negative control, 10 µL of the antibiotic diluent alone was simultaneously added to separate respective wells in the 96-well assay plate. Bacteria and antibiotics were incubated statically for 2 h at 37 °C, 5% CO_2_ in a humidified atmosphere. After 2 h, the 96-well plate was sonicated for 2 m in a water bath sonicator to disrupt any bacterial aggregates. Each well was then serially diluted and spread plated on chocolate agar to determine colony forming units (CFU)/mL. Percent survival was calculated by comparing CFU/mL of the diluent alone (‘no-antibiotic’) with the antibiotic treated bacteria. CFU/mL of the antibiotic wells were divided by CFU/mL of the diluent only wells, multiplied by 100, then this value was subtracted from 100 to calculate percent killing. Experiments were performed with 2–3 technical triplicates per assay, and a minimum of three times on separate days.

### RNA isolation and real-time quantitative reverse transcription PCR (qRT-PCR)

RNA isolation and qRT-PCR was conducted as previously described^36^ with a few modifications. Briefly, to prepare for RNA isolation, 10 cm^2^ flat tissue culture tubes were seeded with 2.5 mL NTHI at 2 × 10^5^ CFU/mL. After 16 h incubation at 37 °C, 5% CO_2_, humidified atmosphere, the tubes were gently washed as described in detail above. Tissue culture tubes were treated with 5 µg MsTipMab per 0.8 cm^2^. The tubes were returned to the incubator, gently inverted so the medium covered the biofilm, and allowed to incubate at 37 °C, 5% CO_2_ for 2 h or 6 h. After 2 h or 6 h, the tubes were inverted, and the α-DNABII NTHI NRel (‘2 h NRel’ or ‘6 h NRel’, respectively) collected by pouring into separate Eppendorf tubes. α-DNABII NTHI NRel were centrifuged for 1 m at 16,000×*g* at 4 °C, the supernatant aspirated, then 1 mL TRIzol Reagent (Thermo Fisher Scientific, Cat no. 15-596-026) added to the bacterial pellets. Suspended bacterial solutions were transferred to separate Phasemaker Tubes (Thermo Fisher Scientific, Cat no. A33248), RNA collected following the manufacturer’s instructions, and RNA purified using a Qiagen RNeasy kit (Qiagen, Cat no. 74106). Residual DNA was removed via treatment with DNase I (NEB, Cat no. M0303L) and SUPERase In RNase Inhibitor (Ambion, Cat no. AM2694) per manufacturer’s instructions for 45 m at 37 °C. DNase I treatment was repeated. qRT-PCR was conducted with the SuperScript III Platinum SYBR Green One-Step qRT-PCR Kit (Invitrogen, Cat no. 11736059), and fold-changes in gene expression calculated via the ΔΔC_t_ method. Primers used for qRT-PCR are listed in Supplemental Table 1. Experiments were performed a minimum of three times with 2–3 technical replicates per assay and on separate days.

### Bacterial killing by human neutrophils

Human neutrophils were isolated from blood via magnetic negative selection using the EasySep Human Neutrophil Isolation Kit (StemCell Technologies, Inc., Cat no. 17957). Susceptibility of NTHI to killing by human neutrophils was assessed as previously described^74^, with a few modifications. 1 × 10^6^ neutrophils were seeded into 1 mL DPBS in a 24-well non-tissue culture treated plate. Neutrophils were activated by addition of 50 nM phorbol 12-myristate 13-acetate (PMA) (Sigma-Aldrich, Cat no. P8139) for 10 m at 37 °C, 5% CO_2_ in a humidified atmosphere. NRel were collected from tissue culture tubes after 2 h or 6 h, as described previously. All bacterial suspensions were sonicated for 2 m in a water bath sonicator to disrupt any aggregates, then diluted such that a range of 4.0 × 10^3^–2.5 × 10^5^ CFU NTHI/1 mL DPBS would be added per well in the 24-well assay plate. For experiments that utilized the intracellular NADPH-oxidase inhibitor, diphenyleneiodonium chloride (DPI) (Sigma-Aldrich, Cat no. D2926), 0.05 µM DPI was added to non-activated neutrophils, allowed to incubate for 30 s at room temperature, then 50 nM PMA added to activate neutrophils, as above^51,74^. Regardless of whether neutrophils were or were not pre-treated with DPI, planktonic or α-DNABII NTHI NRel and activated neutrophils were incubated for 30 m at 37 °C, 5% CO_2_ in a humidified atmosphere. After 30 m, 100 µL 10 × TrypLE (Thermo Fisher Scientific, Cat no. A1217701) was added to each well, vigorously pipetted, diluted, and plated on chocolate agar for enumeration. Experiments were performed with 2–3 technical triplicates per assay on individual days for a minimum of six separate days.

### Assessment of bacterial membrane permeability in NRel populations of NTHI 86-028NP, *Escherichia coli* UTI89*, Pseudomonas aeruginosa* 142-1, or MRSA isolates

Planktonically grown and α-DNABII NTHI, *E. coli* UTI89, *P. aeruginosa* 142-1, or MRSA NRel were assessed for relative membrane permeabilities via use of the fluorescent intercalating dye SYTOX Green Nucleic Acid Stain (Invitrogen, Cat no. S7020)^75,76^. Mid-log phase planktonically grown bacteria were prepared as follows. NTHI from a chocolate agar plate, *E. coli* UTI89 from an LB agar plate, or *P. aeruginosa* 142-1 or MRSA colonies from a TSA plate were separately suspended in 1.5 mL equilibrated respective medium to OD_490_ 0.10, then allowed to grow statically for 3 h (for NTHI or MRSA) or 2.5 h (for *E. coli* UTI89 or *P. aeruginosa* 142-1) with a vented cap at 37 °C with 5% CO_2_. After incubation, the OD_490_ of the planktonically grown culture was read. All bacterial suspensions were centrifuged at 14,000 rpm for 3 m at 4 °C. Planktonically grown bacteria were then resuspended in 900 µL DPBS. α-DNABII NTHI, *E. coli* UTI89, *P. aeruginosa* 142-1, or MRSA NRel were resuspended in 1.2 mL DPBS and centrifuged as before. This process was repeated up to two additional times for α-DNABII NRel to eliminate any background fluorescence.

For 2 h α-DNABII NTHI, *E. coli* UTI89, *P. aeruginosa* 142-1, or MRSA NRel, bacteria were resuspended in a final volume of 900 µL DPBS. 6 h α-DNABII NTHI NRel were resuspended in a final volume of 500 µL DPBS. 6 h NRel were resuspended in a lower volume of DPBS given that CFU/mL of collected bacteria after 6 h biofilm exposure to α-DNABII was less than at 2 h. All bacterial suspensions were gently sonicated for 2 m in a water bath sonicator to disrupt bacterial aggregates. 100 µL aliquots of each bacterial suspension was added to their respective wells in a black MaxiSorp FluoroNunc 96-well plate (Thermo Fisher Scientific, Cat no. 437111) along with 0.5 µM SYTOX Green or 0.5% Triton X-100 (Sigma-Aldrich, Cat no. T8787) plus 0.5 µM SYTOX Green for NTHI and MRSA and 5 µM SYTOX Green or 5 µM SYTOX Green plus 0.5% Triton X-100 (final concentrations) for *E. coli* UTI89 and *P. aeruginosa* 142-1. Use of a greater concentration of SYTOX Green with *E. coli* UTI89 and *P. aeruginosa* 142-1 was necessary as larger genome sizes (compared to MRSA or NTHI) require a greater concentration of SYTOX Green to maximize intercalation across the larger genome and retain linear range of fluorescence^77,78^. Each bacterial suspension was added to respective wells such that a range of 2- to 4 × 10^7^ CFU all respective pathogens/well was achieved. Experiments were performed a minimum of three times on separate days with three technical triplicates per assay. Fluorescence was measured spectrophotometrically (at an excitation wavelength of 480 nm and an emission wavelength of 522 nm) every 15 m for 120 m at 37 °C via FLUOstar Omega microplate reader (BMG LABTECH, Ortenberg, Germany).

### Quantification and statistical analyses

Statistical significance was determined with GraphPad Prism Version 9 by Student’s unpaired two-tailed t-test for comparison of means between two groups, two-way ANOVA with Šidák’s correction or mixed-effects analysis for comparison of more than two groups, or repeated measures linear mixed model for comparison of time course data. Description of statistical analysis used can be found in figure legends. All in vitro assays were repeated a minimum of three times on separate days. A *p*-value ≤ 0.05 is indicated by *, a *p*-value of ≤ 0.01 is indicated by **, a *p*-value of ≤ 0.001 is indicated by ***, and a *p*-value ≤ 0.0001 is indicated by ****.

### Supplementary Information

Supplementary Information.

## Data Availability

The datasets used and/or analyzed for the current study will be available from the corresponding author upon reasonable request.
