# Supplementary material for: Nontypeable Haemophilus influenzae released from biofilm residence by monoclonal antibody directed against a biofilm matrix component display a vulnerable phenotype
Source: Sci Rep. 2023 Aug 10;13:12959. doi: 10.1038/s41598-023-40284-5 (PMC10415356; doi:10.1038/s41598-023-40284-5)
Supplement: Supplementary file 1 — Supplementary Information. [file 41598_2023_40284_MOESM1_ESM.pdf]

Nontypeable *Haemophilus influenzae* released

from biofilm residence by monoclonal antibody directed against a biofilm matrix

component display a vulnerable phenotype

Kathryn Q. Wilbanks[1], Elaine M. Mokrzan[1], Theresa M. Kesler[1], Nikola Kurbatfinski[1],

Steven D. Goodman[1,2], and Lauren O. Bakaletz[1,2*]

[1] Center for Microbial Pathogenesis, Abigail Wexner Research Institute at Nationwide Children's Hospital, Columbus, OH 43205, USA

[2] Department of Pediatrics, The Ohio State University College of Medicine, Columbus, OH 43205, USA

*Corresponding author: Lauren O. Bakaletz, Lauren.Bakaletz@nationwidechildrens.org

Supplementary Table 1. Number (CFU/mL) of NTHI released from biofilm residence after indicated period of biofilm-mAb contact time (NTHI NRel) after one DPBS wash or indicated period of media-biofilm contact time after one DPBS wash.

Timepoint (antibiotic killing)	# α-DNABII NTHI NRel (CFU/mL)	# planktonic NTHI within fluids above biofilm (CFU/mL)	Ratio NTHI NRel:planktonic NTHI
1m	3.3e8	2.4e8	1.4:1
5m	4e8	2.6e8	1.5:1
15m	3.5e8	1.9e8	1.8:1
2h	2.8e8	2.1e8	1.3:1
4h	6e8	3.9e8	1.5:1
6h	6.3e8	2.6e8	2.4:1

Supplementary Table 2. Primers used in this study to determine relative transcript abundances via qRT-PCR.

Primer	Sequence
fis-forward	TAATCCTGCCGATGCCTTAAC
fis-reverse	CGGGTTTGATTACCACGAGTAT
deaD-forward	TGTGGTGAACTACGACATTCC
deaD-reverse	GATCCTGATCGGCTGTGAATAA
artM-forward	GTCTTATCCAATGCGTGGTTCT
artM-reverse	GGATGCTAATGCCGTTCCTTTA
ompP2-forward	AGCACAAGAGCGTCATAAGT

ompP2-reverse	CTGAACGCCATTGCTGATTT
ftsI-forward	CGTGGTGGGTTATACGGATATT
ftsI-reverse	ACAGTACGTGAACCGTCTTTAC
dacA-forward	GGTGCGCATATTATTCGTGATTT
dacA-reverse	AGTCCATTACGGTTAGCTTGTT
dacB-forward	GGCTCTAACGCAGGTGTTATT
dacB-reverse	GTACTCGCAGGCAGCATAAA
acrA-forward	GGAACAGAAATTGTGCGTGTAG
acrA-reverse	CGCGCTGACCGATATGTAAT
acrG-forward	TTAGTGCGTTTGCGTGATATTG
acrG-reverse	TGTCGAGGTTGGATTGATGG
tolC-forward	CGTATTACCACGGCTCTCAATAA
tolC-reverse	CGCTGATTGTAACTATGGGTTTG
acrR-forward	CGGCGATAAATTTAGCCTCTGA
acrR-reverse	TGAATCGCACGCCAAGAG
bla-forward	GTCGCCGCATACACTATTCT
bla-reverse	GCAGCACTGCATAATTCTCTTAC
hktE-forward	GCGATTGAACGTGGTGATTTC
hktE-reverse	TCGGCCACACTTTGGTTAG
pdgX-forward	GCACTCGTCAGGGTGATAAA

pdgX-reverse	GATGAGCAAGTTGGAGTGAATG
sodA-forward	TTCAGGTTGGGCATGGTTAG
sodA-reverse	CCTGCCACTTCTTTACCCATTA
16S-forward	AAAGGAGACTGCCAGTGATAAA
16S-reverse	CCCTCTGTATACGCCATTGTAG

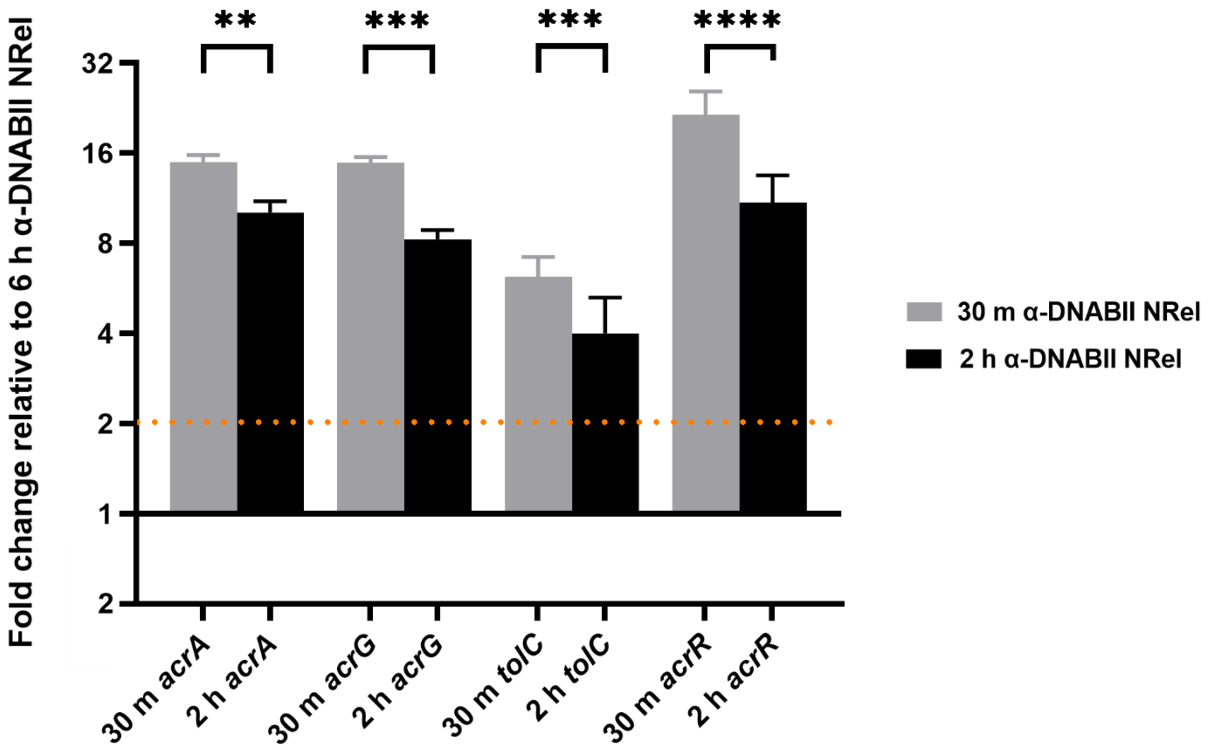

Supplementary Figure 1. Relative transcript abundance of genes that encode the AcrAB-TolC multidrug efflux pump when recovered at 30m or 2h compared to recovery at 6h.

Relative to 6h α-DNABII NTHI NRel, transcript abundance of *acrA*, *acrG*, and *tolC*, the three genes that encode the AcrAB-TolC multidrug efflux pump (MDEP), all exhibit significantly ($p \leq 0.01$ or $p \leq 0.001$) decreased transcript abundance at 2h compared to at 30m. Similarly, transcript abundance of the MDEP repressor, *acrR*, was highly significantly decreased at 2h compared to 30m ($p \leq 0.0001$). These data did not provide additional insight into the observed significant sensitivity of α-DNABII NTHI NRel to killing by A/C that was detected within 5m of biofilm exposure to MsTipMab and maintained for ~6h, by which this sensitivity was no longer detectable. Statistical significance comparing 30m to 2h transcript abundances for each separate gene were independently conducted via two-tailed Mann-Whitney test. Experiments were performed on separate days at least three times with 2-3 technical replicates per assay. Data are represented as mean + SEM.
